# Anatomical Alterations of the Visual Motion Processing Network in Migraine with and without Aura

**DOI:** 10.1371/journal.pmed.0030402

**Published:** 2006-10-17

**Authors:** Cristina Granziera, Alexandre F. M DaSilva, Josh Snyder, David S Tuch, Nouchine Hadjikhani

**Affiliations:** 1 Athinoula A. Martinos Center for Biomedical Imaging, Massachusetts General Hospital, Harvard Medical School, Charlestown, Massachusetts, United States of America; 2 Division of Health Sciences and Technology, Harvard-Massachusetts Institute of Technology, Cambridge, Massachusetts, United States of America; University of Hamburg, Germany

## Abstract

**Background:**

Patients suffering from migraine with aura (MWA) and migraine without aura (MWoA) show abnormalities in visual motion perception during and between attacks. Whether this represents the consequences of structural changes in motion-processing networks in migraineurs is unknown. Moreover, the diagnosis of migraine relies on patient's history, and finding differences in the brain of migraineurs might help to contribute to basic research aimed at better understanding the pathophysiology of migraine.

**Methods and Findings:**

To investigate a common potential anatomical basis for these disturbances, we used high-resolution cortical thickness measurement and diffusion tensor imaging (DTI) to examine the motion-processing network in 24 migraine patients (12 with MWA and 12 MWoA) and 15 age-matched healthy controls (HCs). We found increased cortical thickness of motion-processing visual areas MT+ and V3A in migraineurs compared to HCs. Cortical thickness increases were accompanied by abnormalities of the subjacent white matter. In addition, DTI revealed that migraineurs have alterations in superior colliculus and the lateral geniculate nucleus, which are also involved in visual processing.

**Conclusions:**

A structural abnormality in the network of motion-processing areas could account for, or be the result of, the cortical hyperexcitability observed in migraineurs. The finding in patients with both MWA and MWoA of thickness abnormalities in area V3A, previously described as a source in spreading changes involved in visual aura, raises the question as to whether a “silent” cortical spreading depression develops as well in MWoA. In addition, these experimental data may provide clinicians and researchers with a noninvasively acquirable migraine biomarker.

## Abstract 2

A structural abnormality in the network of motion-processing areas could account for, or be the result of, the cortical hyperexcitability seen in people who have migraine.

## Editors' Summary

**Background.**

Migraine is a disabling brain disorder that affects more than one in ten people during their lifetimes. It is characterized by severe, recurrent headaches, often accompanied by nausea, vomiting, and light sensitivity. In some migraineurs (people who have migraines), the headaches are preceded by neurological disturbances known as “aura.” These usually affect vision, causing illusions of flashing lights, zig-zag lines, or blind spots. There are many triggers for migraine attacks—including some foods, stress, and bright lights—and every migraineur has to learn what triggers his or her attacks. There is no cure for migraine, although over-the-counter painkillers can ease the symptoms and doctors can prescribe stronger remedies or drugs to reduce the frequency of attacks. Exactly what causes migraine is unclear but scientists think that, for some reason, the brains of migraineurs are hyperexcitable. That is, some nerve cells in their brains overreact when they receive electrical messages from the body. This triggers a local disturbance of brain function called “cortical spreading depression,” which, in turn, causes aura, headache, and the other symptoms of migraine.

**Why Was This Study Done?**

Researchers need to know more about what causes migraine to find better treatments. One clue comes from the observation that motion perception is abnormal in migraineurs, even between attacks—they can be very sensitive to visually induced motion sickness, for example. Another clue is that aura are usually visual. So could brain regions that process visual information be abnormal in people who have migraines? In this study, the researchers investigated the structure of the motion processing parts of the brain in people who have migraine with aura, in people who have migraine without aura, and in unaffected individuals to see whether there were any differences that might help them understand migraine.

**What Did the Researchers Do and Find?**

The researchers used two forms of magnetic resonance imaging—a noninvasive way to produce pictures of internal organs—to examine the brains of migraineurs (when they weren't having a migraine) and healthy controls. They concentrated on two brain regions involved in motion processing known as the MT+ and V3A areas and first measured the cortical thickness of these areas—the cortex is the wrinkled layer of gray matter on the outside of the brain that processes information sent from the body. They found that the cortical thickness was increased in both of these areas in migraineurs when compared to healthy controls. There was no difference in cortical thickness between migraineurs who had aura and those who did not, but the area of cortical thickening in V3A corresponded to the source of cortical spreading depression previously identified in a person who had migraine with aura. The researchers also found differences between the white matter (the part of the brain that transfers information between different regions of the gray matter) immediately below the V3A and MT+ areas in the migraineurs and the controls but again not between the two groups of migraineurs.

**What Do These Findings Mean?**

This study provides new information about migraine. First, it identifies structural changes in the brains of people who have migraines. Until now, it has been thought that abnormal brain function causes migraine but that migraineurs have a normal brain structure. The observed structural differences might either account for or be caused by the hyperexcitability that triggers migraines. Because migraine runs in families, examining the brains of children of migraineurs as they grow up might indicate which of these options is correct, although it is possible that abnormalities in brain areas not examined here actually trigger migraines. Second, the study addresses a controversial question about migraine: Is migraine with aura the same as migraine without aura? The similar brain changes in both types of migraine suggest that they are one disorder. Third, the abnormalities in areas MT+ and V3A could help to explain why migraineurs have problems with visual processing even in between attacks. Finally, this study suggests that it might be possible to develop a noninvasive test to help doctors diagnose migraine.

**Additional Information.**

Please access these Web sites via the online version of this summary at http://dx.doi.org/10.1371/journal.pmed.0030402.

- The MedlinePlus encyclopedia has several pages on migraine
- The US National Institute of Neurological Disorders and Stroke offers patient information on migraine and other headaches
- The NHS Direct Online contains patient information on migraine from the UK National Health Service
- MAGNUM provides information from The US National Migraine Association
- The Migraine Trust is a UK charity that supports research and provides support for patients
- The Migraine Aura Foundation is a site about aura that includes a section on art and aura

## Introduction

Migraine is a widespread and debilitating disorder and one of the most common disorders of the nervous system, according to the World Health Organization. Prevalence studies estimate that migraine affects 15%–25% of women and 6%–8% of men [1–4]. More than 2.5 million people in North America have at least one day of migraine per week. Despite the public health significance of this problem, the pathophysiology of migraine is not yet fully understood, and no biomarkers are available for the diagnosis.

Migraineurs exhibit abnormalities in motion processing between attacks [5–8]. Differences in sensitivity to temporal and spatial contrast, color, and orientation have been observed, and many migraineurs report abnormal visual discomfort when viewing striped patterns (e.g. [7,9–16]). In addition, migraineurs are unusually susceptible to visually induced motion sickness [17], which is predictive of migraine development [18].

Motion perception is a complex phenomenon involving subcortical (lateral geniculate nucleus [LGN], superior colliculus, and pulvinar [19]) and cortical structures (visual cortical areas MT+ and V3A [20,21]). In humans, area MT+ is located between the lateral and the inferior occipital sulcus in the occipitotemporal region and is characterized by a well-defined pattern of dense myelination and distinguishing architectonic features [22]. Area V3A is located in the transverse occipital sulcus and is specifically activated by coherent motion [20].

Motion perception deficits have frequently been reported in patients with MWA and MWoA, both in the acute phase and in the interictal period [6,7,11,23,24], and abnormal functions of areas MT+ and V3A have been evidenced in migraineurs. Transcranial magnetic stimulation studies have shown that MWA and MWoA patients have a lower phosphene threshold than HCs when transcranial magnetic stimulation is delivered over V1 and over area MT+ [6,23]. Moreover, psychophysical studies show that both groups of migraineurs exhibit a decreased ability to detect coherent motion [7] between attacks.

One of the leading hypotheses in migraine pathophysiology is that the brains of migraineurs are hyperexcitable [6,23,25]. Enhanced neuronal excitation results in increased extracellular K^+^. If the reuptake and other transport processes are not efficient in controlling glutamate release, a wave of cortical spreading depression (CSD) [26] is likely to arise as a consequence of this extracellular K^+^ increase. CSD is a slow, self-propagating wave of neuronal and glial depolarization, followed by long-lasting suppression of neural activity. CSD has been directly demonstrated in humans suffering from brain trauma or cerebral hemorrhage [27].

In MWA, several studies report the presence of changes that can be related to CSD [28,29], and a phenomenon similar to CSD underlies occipital lobe dysfunction during visual aura [28–37]. In a previous study, which used functional imaging to examine patients during visual aura, we found eight characteristics of CSD using continuous functional resonance magnetic imaging recording, including typical propagation velocity, duration, and suppression of activity [29]. These changes started in area V3A in two recorded attacks.

Functional studies point to hyperexcitability in regions of the visual cortex known to be involved in motion processing in patients with both MWA and MWoA [6,7,11,23,38] (but see [39,40]).

In order to investigate the anatomical substrate of these visual processing abnormalities, we used cortical thickness measurements and DTI to characterize the integrity of the gray and white matter (WM) of regions supporting motion perception in migraineurs.

## Methods

### Participants

In total 24 individuals with migraine (12 with MWA and 12 MWoA), and 15 HCs were enrolled in the study. The patients all met the criteria of the International Classification of Headache Disorders (2nd Ed) criteria for the diagnosis of migraine [41]. Demographic characteristics are given in Table 1. All participants with aura suffered from visual aura in at least half of the attacks (the other half were attacks of MWoA). None had their aura/headache systematically on the same side.

**Table 1:**
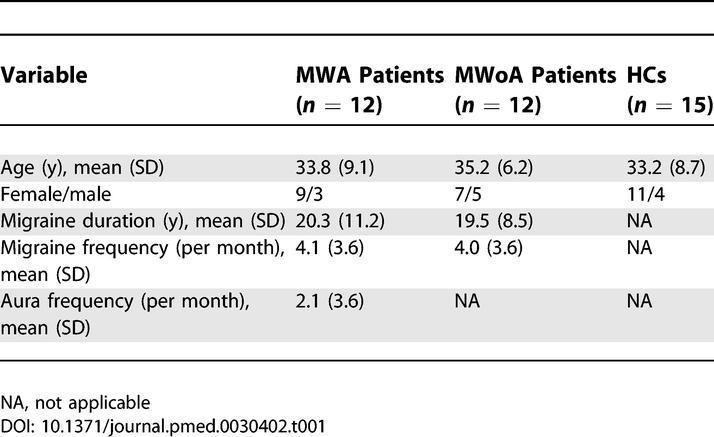
Demographics of Study Participants

Informed written consent was obtained for each participant before the scanning session, and the Massachusetts General Hospital Human Studies Committee approved all procedures under Protocol #2002P-000652.

### Imaging

Two high-resolution (1.0 × 1.0 × 1.25 mm) structural images were obtained with a MP-RAGE (magnetization-prepared rapid acquisition with gradient echoes) sequence, (128 slices; 256 × 256 matrix; echo time (TE) = 3.44 ms; repetition time (TR) = 7.25 ms; flip = 7°) on a 3.0 Tesla Allegra MR scanner (Siemens, Erlangen, Germany). The two scans were motion-corrected with AFNI and averaged to create a single image volume with a high contrast-to-noise ratio.

Whole-head DTI scans were acquired (TR/TE = 8,300/89 ms; slice thickness = 2 mm (0 mm gap); 60–64 slices total; FOV 256 × 256 mm; 128 × 128 matrix; 1 average; 60 directions of encoding with b value = 700 s/mm^2^; and 10 images with b-value = 0 s/mm^2^). Head motion was minimized by the use of tightly padded clamps attached to the head coil. Data were processed using the Freesurfer package.

### Cortical Thickness Analysis

For cortical thickness measurements, we used a technique of analysis developed in our center by Fischl et al. [42]. This automated method accurately measures the thickness of the cerebral cortex across the entire brain and generates cross-individual statistics in a coordinate system based on cortical anatomy. The interindividual standard deviation (SD) of the thickness measures is less than 0.5 mm, implying the ability to detect focal atrophy in small populations or even individual participants. The reliability and accuracy of this new method has been assessed by within-participant test-retest studies, as well as by comparison of cross-participant regional thickness measures with published values. This technique has also been validated with histological and with manual [43,44] measurements.

The maps of cortical thickness were created using previously described procedures [42]. Regions of interest on the cortical surface were defined by detection of contiguous regions of statistical significance (*p* < 0.05) in the maps described above. These regions of interest were created on a standard brain and mapped back to each individual using spherical morphing to find homologous regions across participants. Mean thickness scores for areas MT+ and V3A were calculated for each person. These scores were used to perform unpaired t-test and Bonferroni-corrected ANOVAs between groups for each region of interest.

### DTI Analysis

DTI has emerged as a powerful method for the investigation of WM architecture. The technique measures the tensor associated with the self-diffusion (Brownian motion) of the endogenous water in brain tissue [45]. It is more sensitive to WM alteration than conventional T1 or T2 imaging. Diffusion anisotropy, qualified with the fractional anisotropy (FA) metric [46], is influenced by several factors, including the degree of myelination, density, diameter distribution, and orientational coherence of axons [47]. Deep WM lesions (seen on T1 or T2 images), including small infarcts, have been described in migraineurs [48], although no exploration of more subtle WM changes has been performed.

The diffusion tensor, associated eigensystem, and FA metric were calculated for each voxel [49,50]. The FA volumes were normalized to MNI (Montreal Neurological Institute) space manually. The MNI normalization was performed by registering the individual T2 volume to the MNI-152 T2 template and then applying the registration transformation to the FA volume. The MNI-normalized FA volumes were then smoothed using a three-dimensional Gaussian kernel with 3mm FWHM to improve anatomic correspondence across volumes. Statistical maps for group differences in FA were then computed, using a two-tailed t-test on a voxel-by-voxel basis.

## Results

**Figure 1:**
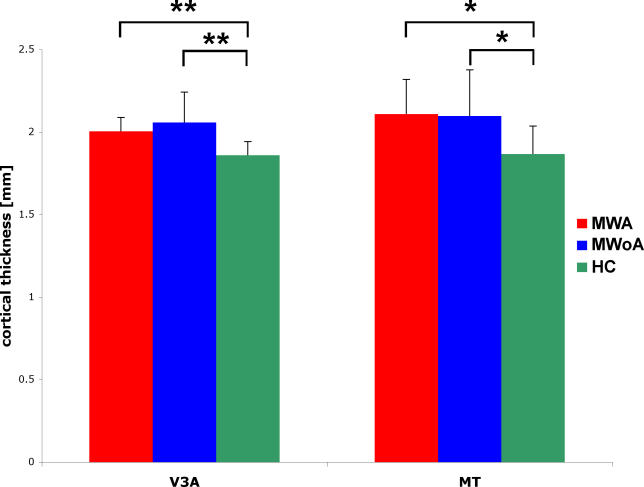
Cortical Thickness Is Increased in Motion-Processing Areas in Migraineurs Values are presented in mean millimeters (SD). V3A: MWA, 2.00 (0.09); MWoA, 2.06 (0.19); HCs, 1.86 (0.08). MT+: MWA, 2.11 (0.21); MWoA, 2.10 (0.88); HCs, 1.87 (0.17). Asterisks represent *p*-value summary * *p* < 0.05; ** *p* < 0.01

An increase of cortical thickness was found bilaterally in two areas involved in motion processing, area V3A and MT+, in migraineurs compared with controls. Values are expressed, in millimeters, as mean (SD) V3A**:** MWA, 2.00 (0.09); MWoA, 2.06 (0.19); HC, 1.86 (0.08). MT+: MWA, 2.11 (0.21); MWoA, 2.10 (0.28); HC, 1.87 (0.17). Unpaired t-tests revealed that both areas were significantly thicker in all migraineurs compared with HCs: V3A, *p* = 0.0002; MT+, *p* = 0.002. One-way ANOVA showed significant differences in V3A and MT+: V3A, *p* = 0.0005; MT+, *p* = 0.010. Bonferroni multiple comparisons test revealed significant differences between each subgroup of migraineurs and HCs (MWA versus HCs: V3A, t = 2.99, *p* < 0.05; MT+, t = 2.82, *p* < 0.05; MWoA versus HCs: V3A, t = 4.12, *p* < 0.001; MT+, t = 2.68, *p* < 0.05) but no significant difference between migraineurs (MWA versus MWoA: V3A, t = 1.08, *p* > 0.05; MT+: t = 0.13, *p* > 0.05) (Figure 1).

**Figure 2:**
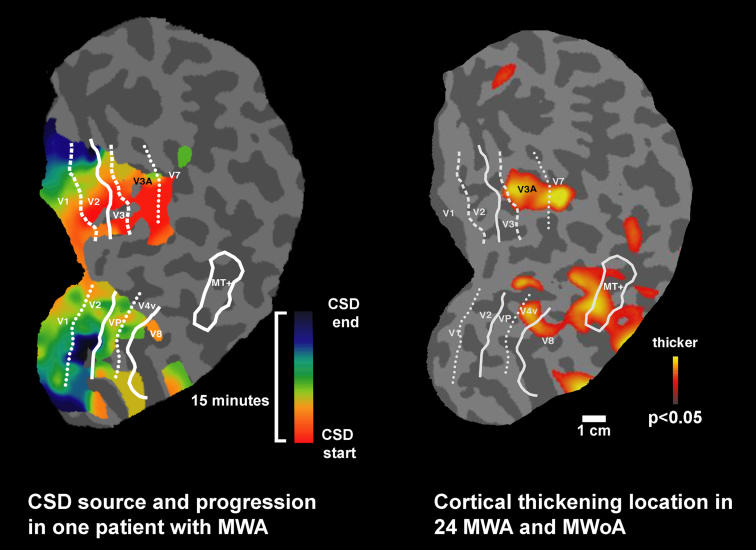
Retinotopic Localization of Cortical Thickness Changes Flattened maps of the right occipital cortex, gyri, and sulci are indicated as light and dark gray. The borders of retinotopic areas are indicated in white (horizontal meridians, solid lines; upper vertical meridians, dotted lines; lower vertical meridians, dashed lines). The image on the left is taken from our previous data [29] and shows the progression of CSD during a visual aura, starting in area V3A, in a single participant. The image on the right shows the average map of the mean thickness difference of 24 migraineurs compared with 15 matched controls, projected on the same brain, with the superimposed retinotopy for one participant. A clear correspondence can be seen between the area of CSD origin in V3A in the left image and cortical thickness difference in the right image. In addition, areas of thickening can be observed in visual area MT+.

Similarity was found between the localization of cortical thickening in the mean thickness difference map of the 24 migraineurs compared with controls in V3A, and the location of the source of a CSD event in a single individual described in our previous work (Figure 2) [29].

**Figure 3:**
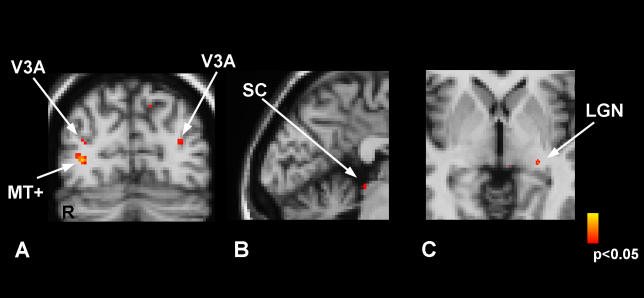
Fractional Anisotropy Differences in Migraineurs Versus Healthy Controls Coronal (A) and sagittal (B) sections show the areas exhibiting statistically significant lower FA values in migraineurs compared to HCs. Significant differences can be seen in the WM underlying MT+ and V3A areas (A), in the superior colliculus (SC) (B), and in the left LGN (C).

FA values in the white matter subjacent to area V3A revealed significantly lower FA bilaterally in the migraine group relative to HC (right hemisphere, *p* = 0.03; left hemisphere, *p* = 0.005). (See Methods for details of analysis.) WM subjacent to area MT+ was lower bilaterally but reached significance only in the right hemisphere (right hemisphere, *p* < 0.001; left hemisphere, *p* = 0.16) (Figure 3A). There was no significant difference between MWA and MWoA subgroups in either area (*p* = 0.5). The superior colliculus and the left LGN, two subcortical areas involved in visual motion perception, also exhibited a significantly lower FA in migraineurs (superior colliculus, *p* = 0.043; LGN, *p* = 0.0035) (Figure 3B and 3C).

## Discussion

This is the first demonstration, to our knowledge, of interictal cortical abnormalities in migraine. Migraine remains an enigmatic disease. The presence of transient neurological signs (aura) in a subgroup of migraineurs has long raised a question that, perhaps surprisingly, still remains controversial: Are MWA and MWoA two separate entities, or are they different manifestations of a similar underlying mechanism? Although the two forms of migraine may be distinct disorders [51,52], similarities between them are remarkable. Pure MWA is comparatively rare, and most MWA patients suffer also from attacks without aura, clearly suggesting a connection between the two forms [53]*.* MWA and MWoA share a number of characteristics: they both have a strong genetic component; they can be triggered by the same substances (usually serotonin releasers such as reserpin or fenfluramine, NO releasers, or high-tyramine foods); both are at higher risks for WM lesions in the cerebellar region of the posterior circulation [54]; and both share the same clinical picture with regard to the pain and related symptoms during the headache phase. In addition, a number of similarities in the therapeutic approach also suggest that a similar pathophysiological phenomenon underlies both disorders—namely, an altered excitability of the cortex. Preventive treatments for both types that have been validated for efficacy in double-blind, placebo-controlled studies include anticonvulsants (divalproex sodium, topiramate); calcium channel antagonists (flunarizine); antidepressants (amitriptyline); beta-blockers (propranolol); and serotonin blocking agents (pizotifen). The two first classes (anticonvulsants, calcium channel antagonists) are likely to reduce cortical excitability, whereas the mechanism of action of three others (antidepressants, beta-blockers, and serotonin blocking agents) is less clear. However, recent data show that migraine prophylactic agents may act by increasing the CSD threshold [55]. A common pathophysiology remains a valid hypothesis, and several recent functional studies have underlined the importance of neuronal hyperexcitability in both types of migraine [25,38].

Our data show that both MWA and MWoA present anatomical changes in precortical and cortical areas involved in motion perception and confirm that these abnormalities are detectable outside the acute phase.

Reduction of anisotropy can be due to several conditions. In the most commonly referred cases, a loss of myelin (as is the case in MS [56–60], stroke [61–63], or in the cerebral arteriopathy CADASIL [64–66]) is accompanied by a loss of barriers to diffusion, and by an increase in mean diffusivity and a decrease in fractional anisotropy. In these patients, decreased performance is correlated with increased mean diffusivity [64], reflecting damage to specific tracts.

Paradoxically, however, a reduced FA can be due to opposite patterns of myelin fiber conditions: loss of myelin, as described above, or increased axonal diameter with increased myelin [67]. In healthy individuals, positive correlations have been described between reaction time in a visuospatial task and visual WM pathways [68]. The faster the individuals were, the lower the FA was. In addition, two studies have shown that intensive training results in lower FA values in the motor pathways of piano players [69,70]. Abnormal white matter maturation processes could result in the alteration observed in motion-processing areas and their subjacent WM, as MT+ is an area that undergoes myelination in an early phase during development [71,72]. This hypothesis cannot be excluded for area V3A, for which no specific developmental data are available presently. Supporting evidence for this interpretation is provided by the early visual processing abnormalities observed in migraineurs, and the close association between motion sickness in childhood and later appearance of migraine [18]. FA changes observed in the WM beneath areas V3A and MT+ may correspond to “thicker” and faster axons, a hypothesis that would be corroborated by the psychophysical findings in migraineurs. Prospective studies in migraineurs' children may help to resolve this question.

An increased thickness of areas V3A and MT+ could be the result of a focal dysplasia leading to exaggerated excitability of neurons in these regions [25,38,73] and to the classical aura percept. In this context, the role of area V3A is particularly intriguing, since it has been suggested as the source of CSD during the aura phase. Our results show that this region is abnormally thickened in both hemispheres in migraine patients, without distinction between subgroups. These findings, as well as the FA abnormalities in V3A supporting white matter, suggest the importance of this area in migraine during the interictal period, as hyperexcitability of the V3A area could contribute to the alteration of motion processing frequently observed in MWA and MWoA.

Most migraine headaches are not preceded by any obvious neurological symptoms, and their origin remains a mystery. Should CSD be of pivotal importance in migraine and aura symptoms reflect a CSD-like phenomenon in the human brain, there is no clear reason that only some migraine patients experience aura. Two imaging studies suggest that MWoA could be the result of a “silent” CSD-like event occurring in the brain: Occipitally originating cortical spreading hypoperfusion was observed by Woods et al. [74] during MWoA; and spreading suppression of neuronal activity in the occipital cortex in MWoA before headache onset was reported by Cao et al. [28]. These data suggest that CSD occurs without being necessarily expressed as aura symptoms and that it could be an underlying phenomenon in both MWA and MWoA.

Alternatively, or in addition, the changes observed may also develop as the consequence of repetitive migraine attacks on the integrity of the cortical layers in V3A and MT+ and the subjacent white matter. The increase in cortical thickness observed in both MWA and MWoA could be due to repeated glial activation following repeated episodes of CSD [75] and/or hypoperfusion [76], known to play a role in migraine pathophysiology [38,77–79].

Neuronal abnormalities in areas MT+ and V3A could explain the common interictal motion-processing deficits observed in both groups (differences in sensitivity to temporal and spatial contrast, color, and orientation; and abnormal visual discomfort when viewing striped patterns [7,9–16]. Following this hypothesis, in addition to these chronic symptoms, migraineurs with aura may present acute episodes of CSD originating from these regions that translate into visual scintillations.

A number of recent studies in migraineurs [54,80] show subclinical structural brain changes, going from progressive cellular damage in nociceptive systems to diffuse WM loss, multifocal WM lesions, and ischemic stroke. However, these changes were present in only a small subset of patients. DTI is more sensitive than conventional T1 and T2 imaging, and can detect subtle white matter changes that are not necessarily ischemic. We did not observe any macroscopic lesions in our participants, but did observe significant FA changes common to all individuals in the migraineurs group that were specifically located in the WM subjacent to areas V3A and MT+ as well as in the superior colliculus and in the lateral geniculate nucleus. We cannot conclude from the present data whether these changes are due to a loss of WM, or to an increase in axonal diameter in these functionally organized areas.

In conclusion, we demonstrate that cortical and subcortical structures involved in motion perception differ between migraineurs and persons without migraine. Chronic dysfunction of these areas may explain interictal findings of motion-processing deficits in migraineurs, whereas episodic dysfunction might contribute to the initiation of an attack. The present data indicate that structural changes are present in at least two cortical regions in migraine patients, even outside attacks; that MWA and MWoA share the same abnormalities; and that the changes are present in areas where CSD most probably develops. It seems likely that MWA and MWoA are two phenotypes sharing a common pathophysiological substrate, and that asymptomatic CSD may occur in some patients.

The finding of morphometric changes in the brains of migraineurs may be of fundamental importance because migraine has so far been considered a condition with abnormal brain function but completely normal brain structure. Further characterization of the histological and chemical properties of V3A, MT+, and the subjacent WM, both prospectively and developmentally, will tell us more about the precise nature of and the mechanisms underlying cortical and subcortical changes in migraineurs.

This study presents the limitation inherent to all question-driven reports, as it voluntary restricted itself to the examination of areas of the brain involved in vision and specifically visual motion processing.

Although our data are experimental and represent the result of a group analysis, the present findings of specific alteration in migraineurs, e.g., cortical thickness increases in specific areas, may open the possibility of a new noninvasive diagnostic tool in migraine.

## Supporting Information

Alternative Language Abstract S1 French Translation of the Abstract (20 KB DOC)

## Abbreviations

CSD: cortical spreading depression
DTI: diffusion tensor imaging
FA: fractional anisotropy
HC: healthy control
LGN: lateral geniculate nucleus
SD: standard deviation
MWA: migraine with aura
MWoA: migraine without aura
WM: white matter
