# Supplementary material for: Anatomical Alterations of the Visual Motion Processing Network in Migraine with and without Aura
Source: PLoS Med. 2006 Oct 17;3(10):e402. doi: 10.1371/journal.pmed.0030402 (PMC1609120; doi:10.1371/journal.pmed.0030402)
Supplement: Supplementary file 1 [file pmed.0030402.sd001.doc]

Abstract - french version

Les patients souffrant de migraine avec ou sans aura présentent des anomalies de la perception visuelle du mouvement aussi bien durant les attaques de migraine qu’entre les crises.

Afin d’investiguer la présence d’un substrat anatomique à ces troubles visuels, nous avons utilisé la résonance magnétique nucléaire et des mesures de l’épaisseur corticale cérébrale, ainsi que l’imagerie du tenseur diffusion (DTI). Nous avons examiné le réseau des aires impliquées dans l’analyse du mouvement visuel chez 24 migraineux (12 avec et 12 sans aura) ainsi que chez 15 sujets contrôles.

Nous avons découvert une augmentation de l’épaisseur du cortex chez les migraineux dans deux aires visuelles impliquées dans l’analyse du mouvement, MT+ et V3A. La substance blanche située directement sous ces deux aires était également anormale. De plus, la DTI a mis en évidence des altérations chez les migraineux de deux autres structures également impliquées dans l’analyse du mouvement: le colliculus supérieur et le corps genouillé latéral.

Une anomalie structurelle du réseau des aires participant à l’analyse du mouvement pourrait être à la base ou être le résultat de l’hyperexcitabilité corticale qu’on observe chez les migraineux. La découverte aussi bien chez les migraineux avec que sans aura d’une augmentation de l’épaisseur corticale dans V3A, aire que nous avons précédemment décrit comme étant la source d’une dépression corticale migrante accompagnant l’aura visuelle, pose la question de la possibilité de la présence de dépression corticale ‘silencieuse’ chez les migraineux sans aura. Finalement, ces découvertes ouvrent la possibilité pour les cliniciens et les chercheurs d’utiliser ces mesures non-invasive comme marqueur biologique de la migraine.
